# Mental and Physical Health in Wilson Disease Patients With SARS‐CoV‐2 Infection and Relevance of Long‐COVID

**DOI:** 10.1002/jmd2.70021

**Published:** 2025-05-06

**Authors:** Isabelle Mohr, Maximilian Brand, Christophe Weber, Andrea Langel, Jessica Langel, Patrick Michl, Viola Yuriko Leidner, Alexander Olkus, Sebastian Köhrer, Uta Merle

**Affiliations:** ^1^ Internal Medicine IV, Department of Gastroenterology University Hospital Heidelberg Heidelberg Germany; ^2^ Internal Medicine III Department of Internal Medicine and Cardiology University Hospital Heidelberg Heidelberg Germany

**Keywords:** COVID‐19, long COVID, mental health, quality of life, SARS‐CoV‐2 infection, Wilson disease

## Abstract

SARS‐CoV‐2 infection and Long COVID (LC) might lead to a significant deterioration of physical and mental health. Wilson disease (WD) patients have a chronic liver and/or neuropsychiatric disease, making it particularly interesting to investigate LC in WD. 51 WD patients were retrospectively examined, evaluating physical and mental health by a survey and neuropsychological tests (SF‐12, PSQI, ISI, Epworth, Chalder‐fatigue scale, PHQ‐9, GAD‐7, PSS, FLei) before and ~11 months after SARS‐CoV‐2 infection. LC was defined as the development of new, at least moderately severe symptoms (shortness of breath, chest pain, fatigue, brain fog, exercise capacity, concentration disturbances) and/or worsening of pre‐existing symptoms. 70.6% had predominant hepatic and 29.4% had neuropsychiatric symptoms at WD diagnosis. Median age was 39 years; 56.1% were female. Patients were in stable maintenance phase with a median treatment duration of 23 years. When compared to before COVID‐19, WD patients had significantly worse physical life quality, sleeping quality, and fatigue. After COVID‐19, a high percentage of WD patients reported concentration disorders (60%), fatigue (55%), reduced exercise capacity (50%), shortness of breath (40%), chest pain (20%) and feeling of brain fog (15%). 39.2% (*n* = 20) of the WD patients were classified as LC. This LC‐WD subgroup showed significantly impaired quality of life, a high stress level, and sleeping disturbances, fatigue, depression, anxiety, and cognitive impairment. A large proportion of WD patients experience LC symptoms, reduced life quality, and sleeping disorders after SARS‐CoV‐2 infection. WD patients post‐infection should be well monitored and supported if they develop persisting symptoms or neuro‐psychological problems.

**Abbreviations**
Chalder: Chalder Fatigue Scale
COVID‐19: SARS‐CoV‐2 infection
Epworth: Epworth Sleepiness Scale
FLei: Fragebogen zur geistigen Leistungsfähigkeit/questionnaire of cognitive performance
GAD‐7: Generalized Anxiety Disorder Scale‐7
ISI: Insomnia Severity Index
PHQ‐9: Patient health questionnaire
PSQI: Pittsburgh sleep quality index
PSS: Perceived stress scale
SF‐12: 12‐item Short Form
WD: Wilson disease

**Summary**

- Patients with Wilson disease show a higher prevalence of Long‐Covid and persistent mental and physical health impairments after SARS‐CoV‐2 infection, highlighting the need for close monitoring and multidisciplinary therapeutic approaches to support recovery and treatment adherence.

## 1 Introduction

Wilson disease (WD) is a rare genetic disorder which results in accumulation of copper in the liver and extrahepatic tissues [1, 2, 3] with a broad severity of symptoms [4, 5]. Almost all patients show evidence of liver disease ranging from increased transaminases to cirrhosis. Neurological and psychiatric symptoms are common in WD [6]. WD is associated with psychological comorbidities with an estimated prevalence of 30%–40% [7] including mood disorders, cognitive impairment, psychosis, anxiety and personality changes [8]. During the pandemic, mental health issues became evident with increased levels of anxiety, stress, and depression compared to pre‐pandemic conditions in general population as well as in patients with chronic diseases [9, 10, 11]. Prolonged symptoms can occur after acute COVID‐19 illness named “long COVID”(LC) with a conservative estimate of global prevalence of ~4% [12]. LC summarizes a heterogeneous, multisystem condition that occurs after SARS‐CoV‐2 infection, characterized by symptoms that persist for at least 12 weeks and cannot be explained by an alternative diagnosis [13]. Symptoms may be new‐onset following recovery or persist from the acute phase and often fluctuate or relapse over time, affecting daily functioning and is recognized to occur regardless of initial infection severity, including also mild or asymptomatic cases [13]. LC symptoms can be of physical and psychological nature ranging from mild to debilitating [14, 15]. Commonly reported symptoms include fatigue, shortness of breath, cough, joint and muscle pain, headaches, sleep disturbances, concentration disorders, chest pain, anxiety, depression and post‐exertional malaise [11, 14, 15, 16]. Diagnosis of LC is primarily based on medical history and the exclusion of other causes. Risk factors may include severe acute COVID‐19 illness or pre‐existing conditions of chronic diseases, female gender, socioeconomic factors and older age [13, 17]. Many studies have sought to identify mechanisms leading to LC and there are likely multiple, potentially overlapping causes including immune dysregulation [13, 18], persisting reservoirs of SARS‐CoV‐2 in tissues [19], autoimmunity [13, 16], microvascular blood clotting [20] and activation of the innate immune defense complement system [21]. WD patients with liver disease and especially those with cirrhosis are at increased risk when infected by SARS‐CoV‐2 due to cirrhosis‐associated immune dysfunction [22]. Treatment of LC is symptomatic and can vary depending on individual complaints [17]. Particularly neuropsychiatric symptoms show a longer symptom duration compared to physical complaints [23]. The majority of LC cases do often not recover in the second year [24], leading to an urgent need for research, especially with regard to LC and its impact on quality of life in ‘at risk disease’ populations. To date, the effects of SARS‐CoV‐2‐infection on liver function and neuropsychological symptoms in WD remain unclear. Our study aims to examine the impact of COVID‐19 and its associated consequences in WD as an example for a hepatic and neuropsychological chronic disease with focus on mental health and the occurrence of LC in these patients.

## 2 Methods

### 2.1 Study Cohort and Conception

**FIGURE 1:**
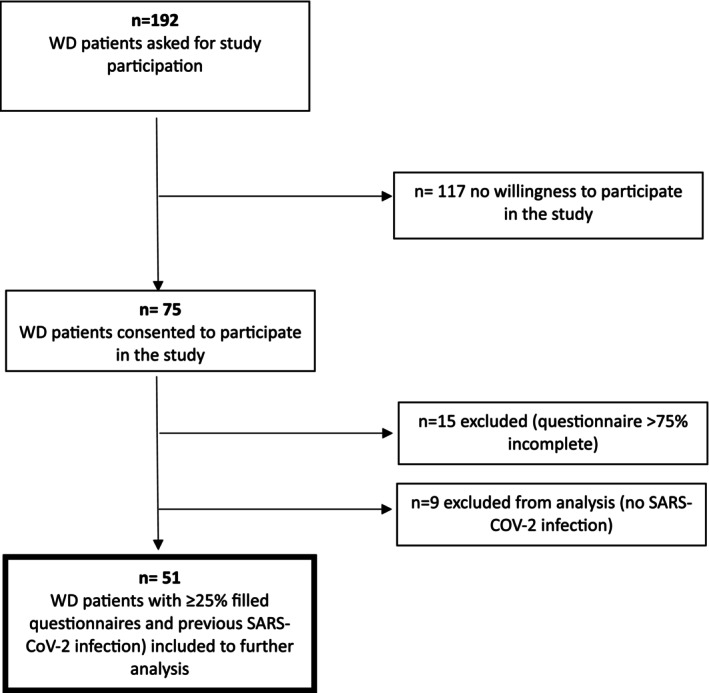
Inclusion criteria.

All adult WD patients visiting our outpatient clinic between January and April 2023 were invited to participate in this post‐COVID‐19 study. In May 2023, 192 patients with confirmed WD (Leipzig score ≥ 4) received the survey by mail. Responses were collected from May to July 2023, with a 39% return rate. Of 75 respondents, 60 completed the survey, and 51 COVID‐19‐infected patients were analyzed. Figure 1 outlines the inclusion criteria. The paper‐based survey retrospectively assessed COVID‐19 illness, vaccination history, new or worsening symptoms, and mental health status. LC was defined as the development of new symptoms or the worsening of pre‐existing symptoms (in the fields of shortness of breath, chest pain, fatigue, brain fog, exercise capacity and concentration disturbances) in at least moderately intense severity and persistence of these till the timepoint of answering the survey. Symptoms already present before COVID‐19 onset and that did not worsen during or after COVID‐19 were not quoted as LC. Patients were asked to rate all symptoms also in retrospect for the situation before their COVID‐19 illness. Ethics approval was obtained (University of Heidelberg, protocols S‐565/2011 and S‐546/2020), and all participants provided written informed consent.

### 2.2 Materials and Measures

#### 2.2.1 Vaccination Status and SARS‐CoV‐2 Infection(s)

In the general part of the survey, patients were asked to notice their dates of SARS‐CoV‐2 vaccination. Additionally, patients were asked about previous SARS‐CoV‐2 infection(s).

#### 2.2.2 Symptom Questionnaire

Patients were asked for current symptoms, if these symptoms were present already before COVID‐19 or if they newly occurred or aggravated during or after acute COVID‐19. If symptoms were present, the severity of each symptom was assessed as either mild, moderate, or severe (Table S2).

#### 2.2.3 Physical and Mental Health Assessments

Physical and mental health were assessed by a survey including different neuropsychological tests before and ~11 months after SARS‐CoV‐2 infection.

#### 2.2.4 Life Quality Assessment via the 12‐Item Short Form (SF‐12)

The 12‐item short form survey (SF‐12) [25] is a compact health questionnaire used to assess an individual's overall health‐related quality of life by measuring physical and mental health aspects within 12 items. Each scale (physical and mental health) score ranges from 0 to 100, with higher scores indicating a better health condition [26].

#### 2.2.5 Pittsburgh Sleep Quality Index (PSQI)

The PSQI evaluates quality of sleep, consisting of 19 self‐rated questions and 5 questions that are to be assessed by a partner/housemate, if available. Only the self‐rated questions are included in the evaluation [27]. The 19 self‐rated questions are combined into 7 components. Each component can have a value between 0 and 3 points. The 7 components are summed to obtain the total score (0–21). The total PSQI score can range from 0 to 21 points. Healthy sleepers typically have a total score of no more than 5 points. Poor sleepers usually have scores between 6 and 10 points. Chronic sleep disorders are generally indicated by scores higher than 10 points [28].

#### 2.2.6 Insomnia Severity Index (ISI)

ISI is designed as a brief screening tool for insomnia. The seven‐item questionnaire asks respondents to rate the nature and symptoms of their sleep problems [29]. Questions relate to subjective qualities of the respondent's sleep, including the severity of symptoms, the respondent's satisfaction with his or her sleep patterns, the degree to which insomnia interferes with daily functioning, how noticeable the respondent feels his or her insomnia is to others, and the overall level of distress created by the sleep problem using Likert‐type scales. Responses can range from 0 to 4, where higher scores indicate more acute symptoms of insomnia. A total score of 0–7 indicates “no clinically significant insomnia,” 8–14 means “subthreshold insomnia,” 15–21 is “clinical insomnia (moderate severity),” and 22–28 means “clinical insomnia (severe)” [29].

#### 2.2.7 Epworth Sleepiness Scale

The standardized questionnaire is intended to assess daytime sleepiness [30]. The questionnaire retrospectively assesses an individual's subjective estimation of the likelihood of falling asleep in eight given typical situations on an ascending rating scale from 0 to 3. The results of the eight questions are summed up to a single score. A score of 0–9 is considered normal, while higher scores indicate severe daytime sleepiness (> 15).

#### 2.2.8 Chalder Fatigue Scale

The Chalder Fatigue Scale is a questionnaire used to assess the severity of fatigue, a symptom that occurs in various conditions and in LC [31, 32, 33]. To evaluate the severity of fatigue, the scale includes eleven questions regarding various impacts of fatigue on performance, which can be weighted differently. From the responses, a numerical value can be derived that quantifies the severity of fatigue. The results range from 0 to 33, and lower scores indicate less fatigue, while scores of 4 or above indicate severe fatigue.

#### 2.2.9 Patient Health Questionnaire (PHQ‐9)

PHQ‐9 is a 9‐item self‐reported inventory that evaluates the criteria for Major Depressive Disorder using a Likert‐type scale with responses from 0 (not at all) to 3 (nearly every day) [34]. It is applied in screening, diagnosing, and monitoring the severity of depressive symptoms [35]. The score ranges from 0 to 27, with a total score of 10 or higher typically indicating possible depression [36].

#### 2.2.10 Generalized Anxiety Disorder Scale‐7 (GAD‐7)

GAD‐7 is a short self‐report questionnaire assessing the presence and severity of generalized anxiety disorder symptoms in individuals [37]. The total score, based on seven items, ranges from 0 to 21, with higher scores indicating greater severity of anxiety.

#### 2.2.11 Perceived Stress Scale (PSS)

PSS is a psychological tool designed to evaluate an individual's perception of stress by assessing the perceived stressfulness of life situations [38]. Scores range from 0 to 40, with higher scores indicating greater perceived stress.

#### 2.2.12 FLei

FLei is a questionnaire designed to assess subjective cognitive complaints [39]. The test is self‐administered by the patient, who must respond to 35 statements using a five‐point rating scale (never; rarely; sometimes; often; very often). The answer “never” receives no points, “rarely” one point, “sometimes” two points, “often” three points, and “very often” four points. Questions are focused on difficulties in everyday situations in the last 6 months, examining three cognitive areas: attention (A), memory (M), executive functioning (E). There are also five control questions to check the response tendency (K). Besides the three cognitive domains, the main score “mental ability” is calculated as the sum of the three cognitive domains (0–120 points) [40]. Higher scores and sub‐scores indicate more difficulties in everyday situations among the test fields mentioned above.

#### 2.2.13 Statistical Analysis

For data analysis, the pseudonymized Excel database was exported to IBM SPSS Statistics (Version 27.0). Descriptive statistics, including frequencies, central tendency, and dispersion, were used. Mental health scores before and after COVID‐19 were compared using a 2‐sample t‐test and Cohen's d to detect effect size. A previous sample size calculation based on anticipated effect sizes (Cohen's d 0.5) and desired statistical power (0.80) delivered *n* = 34.

## 3 Results

### 3.1 Patient Demographics and Variety of Symptoms

**FIGURE 2:**
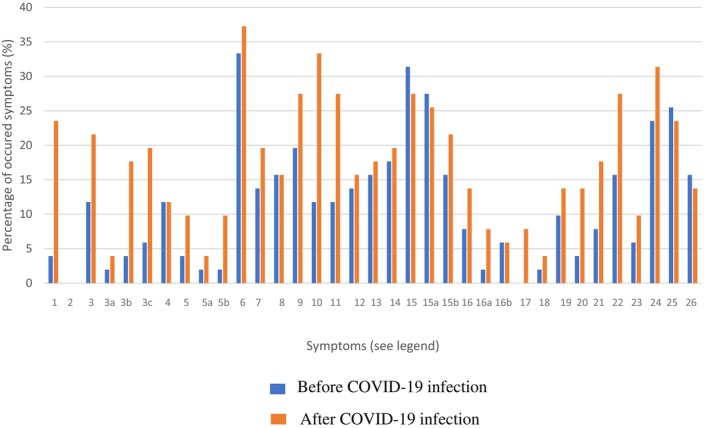
Summary of symptoms before and after SARS‐CoV‐2 infection (%). Before COVID‐19 infection. After COVID‐19 infection. Depicted are symptom frequencies [%] in Wilson disease patients before SARS‐CoV‐2 infection (in retrospect) and at the timepoint the symptom questionnaire was carried out. Symptoms are as follows: 1 Cough, 2 Fever, 3 Shortness of breath (unspecified), 3a Shortness of breath at rest, 3b Shortness of breath at mild activity, 3c Shortness of breath at extended activity, 4 Palpitation, 5 Chest pain (unspecified), 5a Chest pain at rest, 5b Chest pain at extended activity, 6 Fatigue, 7 Myalgia, 8 Arthralgia, 9 Muscular weakness, 10 Cephalgia, 11 Vertigo, 12 Feeling of “brain fog”, 13 Anxiety, 14 Depression, 15 Sleep disturbances (unspecified), 15a Sleep disturbances in falling asleep, 15b Sleep disturbances in sleeping through the night, 16 Stool abnormalities (unspecified), 16a Diarrhea, 16b Constipation, 17 Dysgeusia, 18 Dysosmia, 19 Alopecia, 20 Sore throat, 21 Rhinitis, 22 Reduced physical exercise capacity, 23 Blood pressure variations, 24 Concentration disturbances, 25 Amnestic dysphasia, 26 Memory disturbances.

The study included 51 SARS‐CoV‐2‐infected WD patients, 56.1% female (Table 1), with a mean age of 39 years (SD 15.3). Comparing patients with primarily hepatic manifestation to those with primarily neurologic/psychiatric or mixed manifestation, the onset of symptoms occurred significantly (*p* = 0.001) earlier in primarily hepatic patients (median age at diagnosis 14.7y (SD 9.5)) than in those with primarily neurologic/psychiatric or mixed manifestation (median age at diagnosis 24.5y (SD 9.6)). Hepatic presentation was observed in 70.6%, and 29.4% had neurological symptoms. Liver cirrhosis was present in 13.7% (*n* = 7), with 5 in Child‐Pugh stage A and 2 in stage B. Table S3 presents the genotype among the included phenotypes. The most common mutation in the analyzed cohort was the p.H1069Q mutation (*n* = 47). In our cohort, 8 out of 17 (47.1%) patients with homozygous mutation for H1069Q suffered from a neurological or mixed phenotype. W779X (*n* = 7) and R969Q (*n* = 5) were 2nd and 3rd most common mutations. All patients had at least one SARS‐CoV‐2 infection, and 12% were infected twice. Vaccination rates for 1st and 2nd doses were 92.2%, with 86.3% fully vaccinated at least 2 weeks before infection. All patients with LC symptoms were vaccinated at least twice before infection. The median interval from COVID‐19 illness to evaluation was 10.9 months (range 2.5–40.8; SD 6.0). Figure 2 shows symptom occurrence before and after infection. Fatigue, cephalgia, and concentration disorders were most common, with symptoms generally more frequent post‐infection. Notably, general sleeping disorders decreased, but issues with maintaining sleep increased after COVID‐19.

**TABLE 1:** Patient baseline characteristics.

Parameter	Numbers, medians and percentages (in %)
Number of included WD patients	51 (41.2% male, 58.8% female)
Demographics:	
Age at study inclusion (years)	39 (range 19–70; SD 15.3)
Age at diagnosis (years) total cohort	16 (range 1–50; SD 9.9)
In primarily hepatic phenotype	14 (range 1–50; SD 9.5)
In primarily neurological/mixed phenotype	24 (range 3–37; SD 9.6)
Clinical presentation of WD	*n* = 36 (70.6%) primarily hepatic *n* = 7 (13.7%) primarily neurological *n* = 8 (15.7%) mixed presentation (hepatic + neurological)
Liver cirrhosis	*n* = 7 (13.7%; *n* = 5 CHILD Pugh A, *n* = 2 CHILD Pugh B)
Treatment at study inclusion	D‐penicillamine *n* = 15 (29.4%) Trientine *n* = 35 (68.6%) Zinc *n* = 3 (5.9%; thereof *n* = 2 with additional trientine)
Previous treatment duration (years)	23 (range 2–56; SD 15.4)
Number of SARS‐CoV‐2 infections	*n* = 51 (100%); none hospitalized due to acute COVID‐19
One infection	*n* = 51 (100%)
Two infections	*n* = 6 (12%)
Long‐COVID Symptoms	*n* = 20 (39.2%)
After 1st infection	*n* = 16 (31.4%)
After 2nd infection	*n* = 4 (7.8%)
Time duration of acute COVID‐19 till timepoint of evaluation (months)	10.9 (range 2.5 to 40.8; SD 6.0)
COVID‐19 vaccination	
1st vaccination	*n* = 47 (92.2%)
2nd vaccination	*n* = 47 (92.2%)
3rd vaccination	*n* = 44 (86.3%)

### 3.2 Mental Health Assessments in Whole Cohort

**FIGURE 3:**
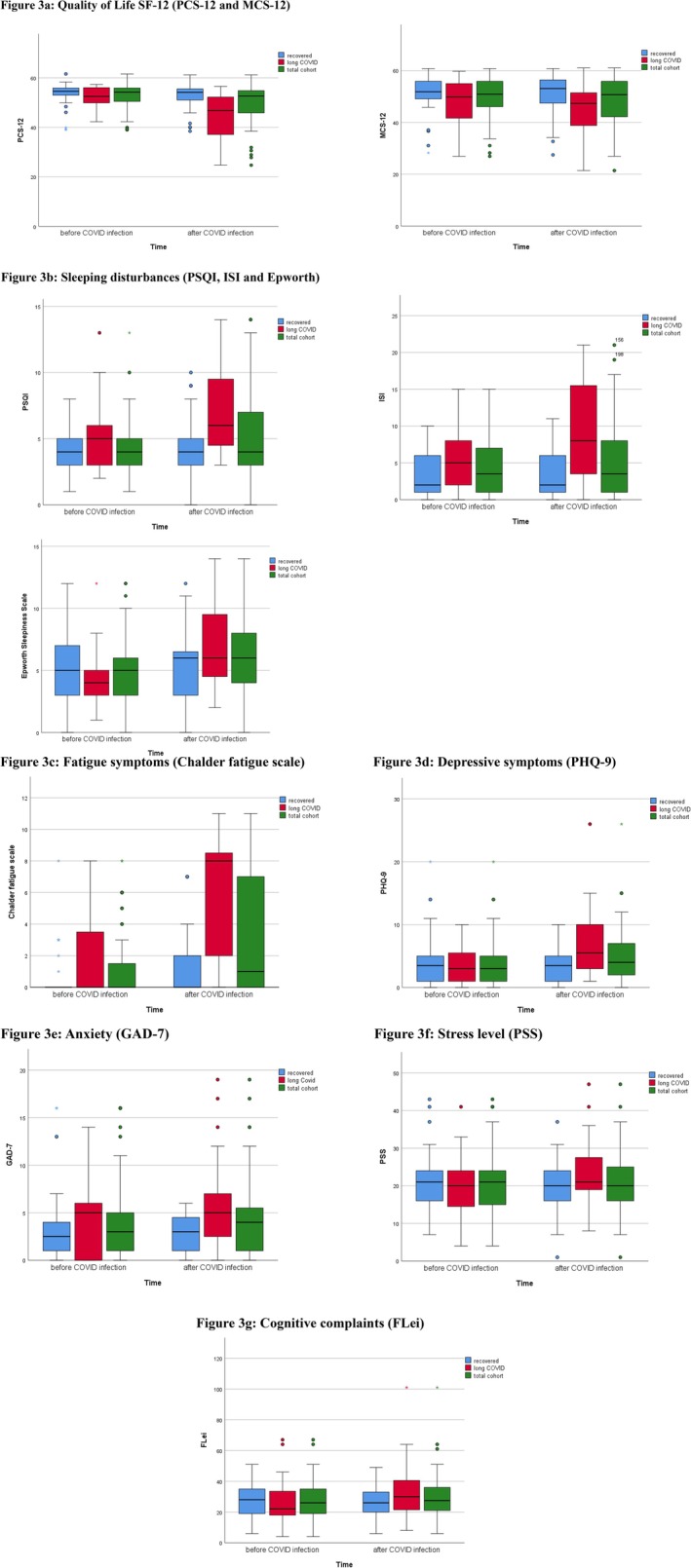
(a–g) Results of neuropsychological tests before and after SARS‐CoV‐2 infection. (a) Quality of Life SF‐12 (PCS‐12 and MCS‐12). (b) Sleeping disturbances (PSQI, ISI and Epworth). (c) Fatigue symptoms (Chalder fatigue scale). (d) Depressive symptoms (PHQ‐9). (e) Anxiety (GAD‐7). (f) Stress level (PSS). (g) Cognitive complaints (FLei). (a–g) Shows the different neuropsychological scores before and after SARS‐CoV‐2 infection. The y‐axis shows the respective absolute value of the applied clinical score. The x‐axis plots the responses of the respective cohorts (total cohort, recovered cohort and Long‐COVID cohort) before and after SARS‐CoV‐2 infection as box‐plots.

Descriptive statistics of mental health assessments (*n* = 51) are summarized in Table 2 and Figure 3a–g. SF12 showed a significantly lower physical health subscore after SARS‐CoV‐2 infection, while the mental health subscore showed no significant change (Figure 3a).

**TABLE 2:** Descriptive statistics of neuropsychological measures before and after acute COVID‐19 (total cohort).

Scoring scale			Before COVID‐19		After COVID 19	95% confidence interval difference (paired)	*t*‐Test	Cohen's d
		*N*	Mean (range, SD)	*N*	Mean (range, SD)	Lower vs. upper value	*p* *	Effect size
Quality of life	SF12‐PHS	51	52.6 (39.0–61.6; 5.1)	51	49.1 (24.7–61.2; 8.6)	1.7 vs. 5.7	**< 0.001**	0.531
	SF12‐MHS	51	49.2 (26.9–60.8; 8.5)	51	48.3 (21.4–61.0; 9.8)	0.3 vs. 2.9	0.100	0.242
Sleeping disturbances	PSQI	51	4.5 (1–13; 2.2)	51	5.4 (0–14; 3.3)	0.2 vs. 1.7	**0.011**	0.600
	ISI	51	4.5 (0–15; 3.8)	51	5.7 (0–21; 5.6)	0.3 vs. 2.5	**0.013**	0.573
	Epworth	51	5.0 (0–12; 2.9)	51	6.1 (0–14; 3.4)	0.4 vs. 1.9	**0.003**	0.645
Fatigue symptoms	Chalder	51	1.2 (0–8; 2.2)	51	3.2 (0–11; 3.8)	1.1 vs. 3.1	**< 0.001**	0.603
Depressive symptoms	PHQ9	51	3.9 (0–20; 4.0)	51	4.9 (0–26; 4.7)	0.2 vs. 2.3	0.105	0.238
Anxiety	GAD7	51	3.8 (0–16; 4.1)	51	4.2 (0–19; 4.1)	0.6 vs. 1.5	0.385	0.122
Stress level	PSS	51	20.70 (4–43; 8.6)	51	21.0 (1–47; 8.7)	1.4 vs. 2.7	0.510	0.095
Cognitive complaints	FLei	51	26.9 (4–67; 14.2)	51	29.5 (6–101; 16.5)	0.1 vs. 4.5	0.060	0.295

Abbreviations: Chalder, Chalder Fatigue Scale; Epworth, Epworth Sleepiness Scale; FLei, Fragebogen zur geistigen Leistungsfähigkeit (questionnaire of cognitive performance); GAD‐7, Generalized Anxiety Disorder Scale‐7; ISI, Insomnia Severity Index; PHQ‐9, Patient health questionnaire; PSQI, Pittsburgh sleep quality index; PSS, Perceived stress scale; SF‐12, 12‐item Short Form.

* *p*‐value significance level < 0.05 (in bold).

Sleep disorders (PSQI, ISI, Epworth; Figure 3b): Scores were more abnormal post‐infection, though most patients had normal results both before and after. Abnormal scores were observed in 3.9% (PSQI > 10), 19.6% (ISI ≥ 8), and 7.8% (Epworth ≥ 10).

Chalder Fatigue Score (Figure 3c) was significantly higher post‐infection, with 11.7% (*n* = 6) scoring ≥ 4, indicating severe fatigue. No significant differences were found for depression, anxiety, stress, or cognitive complaints (PHQ9, GAD7, PSS, FLei; Figure 3d–g; Table 2). Multivariate regression (95% confidential interval) adjusted for LC and possible confounders such as sex (*p* = 0.946), age (0.210), neuropsychiatric manifestation (0,760) and dual vaccination (0.522) did not reveal an independent significant confounder for these effects.

### 3.3 Analysis of Subgroups

The cohort was divided into two groups: LC (*n* = 20) and “recovered” (*n* = 31) (Tables 3 and 4). When analyzing the different phenotypes within the group of LC patients, 15% suffered from primarily neurologic/psychiatric or mixed manifestations, whereas in the “recovered group” even 38% suffered from primarily neurologic/psychiatric or mixed manifestations. Interestingly, 85% of the WD patients with LC suffered from primarily hepatic manifestations. In contrast, only 62% of the “recovered group” suffered from primarily hepatic manifestations. LC was defined as the development or worsening of at least one typical symptom (e.g., shortness of breath, chest pain, fatigue, brain fog, exercise capacity, or concentration disturbances) at moderate intensity, persisting until the survey. Patients without these criteria were classified as recovered. Using this definition, 39.2% (*n* = 20) had LC, while 60.8% (*n* = 31) were considered recovered. Tables S1 and S2 list new symptoms in both subgroups. In the LC group, the most common symptoms were concentration disorders (60%), fatigue (55%), reduced exercise capacity (50%), shortness of breath (40%), chest pain (20%), and brain fog. Significant differences between recovered and LC patients were found for fatigue (*p* = 0.035), exercise capacity (*p* = 0.004), shortness of breath (*p* < 0.001), and concentration disorders (*p* < 0.001). A broad range of new or aggravated symptoms developed post‐infection in the cohort.

**TABLE 3:** Descriptive statistics of neuropsychological measures before and after COVID‐among WD patients with long COVID.

Scoring scale			Before COVID‐19 infection		After COVID 19 infection	95% confidence interval difference	*t*‐Test	Cohen's d
		*N*	Mean (range, SD)	*N*	Mean (range, SD)	Lower vs. upper value	*p* *	Effect size
Quality of life	SF12‐PHS	20	52.3 (42.3–57.3; 4.5)	20	44.6 (24.7–56.6; 10.2)	3.5 vs. 11.9	**0.001**	0.854
	SF12‐MHS	20	47.7 (26.9–59.7; 9.3)	20	45.1 (21.4–61.0; 11.0)	0.4 vs. 5.8	**0.024**	0.545
Sleeping disturbances	PSQI	20	5.1 (2–13; 2.8)	20	7.0 (3–14; 3.7)	0.4 vs. 3.6	**0.014**	0.624
	ISI	20	4.9 (0–15; 4.0)	20	8.6 (0–31; 6.9)	1.0 vs. 5.9	**0.003**	0.660
	Epworth	20	4.5 (1–12; 2.4)	20	7.1 (2–14; 3.5)	0.9 vs. 4.4	**0.004**	0.750
Fatigue symptoms	Chalder	20	1.7 (0–8; 2.6)	20	5.8 (0–14; 4.1)	2.3 vs. 6.1	**< 0.001**	1.063
Depressive symptoms	PHQ9	20	3.8 (0–10; 3.2)	20	7.4 (1–26; 5.9)	1.6 vs. 5.6	**0.001**	0.840
Anxiety	GAD7	20	4.1 (0–14; 3.9)	20	6.4 (0–19; 5.3)	0.5 vs. 3.8	**0.010**	0.623
Stress level	PSS	20	19.9 (4–41; 8.5)	20	24.1 (8–47; 9.6)	1.2 vs. 6.7	**0.005**	0.676
Cognitive complaints	FLei	20	25.8 (4–67; 16.7)	20	34.1 (8–101; 21.6)	2.7 vs. 13.7	**0.005**	0.699

Abbreviations: Chalder, Chalder Fatigue Scale; Epworth, Epworth Sleepiness Scale; FLei, Fragebogen zur geistigen Leistungsfähigkeit (questionnaire of cognitive performance); GAD‐7, Generalized Anxiety Disorder Scale‐7; ISI, Insomnia Severity Index; PHQ‐9, Patient health questionnaire; PSQI, Pittsburgh sleep quality index; PSS, Perceived stress scale; SF‐12, 12‐item Short Form.

* *p*‐value significance level < 0.05 (in bold).

**TABLE 4:** Descriptive statistics of neuropsychological measures before and after COVID‐among WD patients recovered from COVID‐19.

Scoring scale			Before COVID‐19 infection		After COVID 19 infection	95% confidence interval difference	*t*‐Test	Cohens's d
		*N*	Mean (range, SD)	*N*	Mean (range, SD)	Lower vs. upper value	*p* *	Effect size
Quality of life	SF12‐PHS	31	52.9 (38.9–61.6; 5.6)	31	52.2 (38.4–61.2; 5.7)	0.2 vs. 1.7	0.096	0.270
	SF12‐MHS	31	50.2 (28.2–60.8; 7.9)	31	50.7 (27.5–60.8; 8.6)	0.8 vs. 1.8	0.988	0.295
Sleeping disturbances	PSQI	31	4.0 (1–8; 1.7)	31	4.2 (0–10; 2.5)	0.3 vs. 3.6	0.388	0.169
	ISI	31	3.6 (0–10; 3.1)	31	3.4 (0–11: 3.4)	0.5 vs. 2.9	1.0	0.034
	Epworth	31	5.4 (0–12; 3.1)	31	5.3 (0–12; 3.0)	0.3 vs. 2.1	0.103	0.313
Fatigue symptoms	Chalder	31	0.7 (0–8; 1.8)	31	1.2 (0–7; 2.0)	1.0 vs. 3.4	**0.001**	0.326
Depressive symptoms	PHQ9	31	4.1 (0–20; 4.6)	31	3.3 (0–10; 2.6)	0.3 vs. 2.5	0.290	0.204
Anxiety	GAD7	31	3.6 (0–16; 4.4)	31	2.8 (0–6; 2.2)	0.2 vs. 1.6	0.269	0.270
Stress level	PSS	31	21.4 (7–43; 8.7)	31	19.2 (1–37; 7.6)	1.5 vs. 2.6	0.259	0.214
Cognitive complaints	FLei	31	27.1 (6–51; 12.1)	31	25.9 (6–49; 11.1)	0.4 vs. 5.6	0.252	0.217

Abbreviations: Chalder, Chalder Fatigue Scale; Epworth, Epworth Sleepiness Scale; FLei, Fragebogen zur geistigen Leistungsfähigkeit (questionnaire of cognitive performance); GAD‐7, Generalized Anxiety Disorder Scale‐7; ISI, Insomnia Severity Index; PHQ‐9, Patient health questionnaire; PSQI, Pittsburgh sleep quality index; PSS, Perceived stress scale; SF‐12, 12‐item Short Form.

* *p*‐value significance level < 0.05 (in bold).

### 3.4 Mental Health Assessments in WD Patients With LC Symptoms

Patients with LC showed more abnormal neuropsychological test results after SARS‐CoV‐2 infection compared to before (Table 3). Tests for quality of life (SF12), sleep disturbances (PSQI, ISI, Epworth), fatigue (Chalder fatigue score), depression (PHQ9), anxiety (GAD7), stress (PSS), and cognitive complaints (FLei) revealed significantly worse scores post‐infection. The LC group (65% female, median age 33 years, SD 15.8) included 25% with neurological symptoms and 15% with compensated liver cirrhosis. All patients were vaccinated twice before 1st infection. Treatments included trientine (80%) and d‐penicillamine (15%), compared to 63.3% and 38.7% in recovered patients. Differences in copper chelation treatments were not statistically significant (Fisher's exact test).

### 3.5 Mental Health Assessments in WD Patients With Recovered From COVID‐19

The descriptive statistics of mental health assessments before and after COVID‐19 infection of WD patients who recovered from COVID‐19 (*n* = 31) are summarized in Table 4. With the exception of the Chalder fatigue scale (*p* = 0.001), none of the assessments reached statistical significance.

## 4 Discussion

This study examines LC approximately 11 months after SARS‐CoV‐2 infection in WD patients, reflecting a typical cohort with hepatic and neurological manifestations [41, 42, 43, 44]. Symptoms and neuropsychological scores worsened post‐infection, with fatigue, cephalgia, and concentration disorders most frequently reported. Fatigue was the most frequently reported symptom followed by cephalgia and concentration disorders. In other studies, commonly reported LC symptoms included fatigue, shortness of breath, sleep disturbances, concentration disorders (often referred to as brain fog), chest pain and anxiety or depression [11, 14, 15, 16]. Our findings in the WD cohort are consistent with these descriptions. A German study [16] found that 22.9% of patients were symptom‐free 12 months after SARS‐CoV‐2 infection, with reduced exercise capacity (56.3%) and fatigue (53.1%) being most common, leading to lower physical and mental quality of life. Our findings on reduced life quality in WD patients with LC, persisting for a median of 11 months, align with Seeßle et al.'s study [16]. LC symptoms in our cohort included concentration disorders (60%), fatigue (55%), reduced exercise capacity (50%), shortness of breath (40%), chest pain (20%), and brain fog (15%). These symptoms in WD patients are similar to those in the general population [13, 15, 16, 45], with LC affecting both physical and psychological health [13, 46, 47, 48]. WD patients often report significant mental health impacts due to chronic liver and neurological diseases in 30%–40% of cases [7]. Zimbrean et al. studied the mental well‐being of WD patients during the COVID‐19 pandemic, finding increased depression and lower self‐perceived mental health post‐pandemic [49]. Our study, however, focused on clinical and neuropsychological functions after a confirmed SARS‐CoV‐2 infection. Similar to Zimbrean et al., we observed cognitive variations in WD patients post‐infection, showing they differ from the general population [49]. While our data indicate reduced physical quality of life nearly a year after SARS‐CoV‐2 infection, Zimbrean et al. found a generally lower quality of life post‐pandemic. It remains unclear whether the reduced quality of life is due to the pandemic or SARS‐CoV‐2 infection, particularly if patients have not fully recovered. WD patients with LC showed a significant decline in both physical and mental health on the SF‐12 after SARS‐CoV‐2 infection. Scores for sleep disturbances (PSQI, ISI, Epworth) and fatigue (Chalder) were significantly worse, reflecting increased complaints in these areas. These findings are underlined by mild to strong effect size measures reflected in Cohen's d. The LC subgroup also reported higher levels of stress and anxiety compared to before infection. In contrast, no significant changes were observed in the recovered WD subgroup, except for fatigue on the Chalder scale. This supports the effectiveness of our LC definition in distinguishing it from the recovered group, where only fatigue showed significant change. Fatigue in WD patients is likely multifactorial, as it is common in chronic liver diseases [50]. A recent estimate puts global LC prevalence at 4% [12]. In our cohort, LC symptoms were present in 39%, a much higher rate than in the general population, with a median duration of 11 months post‐infection. Even if non‐respondents had no LC, the prevalence would still be 10.4%, higher than the general population. Our conservative LC definition likely prevents overestimating its prevalence in WD. Possible risk factors for LC include pre‐existing chronic diseases, female gender, and older age. WD, as a chronic liver and neurological disease, may explain the higher LC prevalence in this cohort compared to the general population [12, 13]. Inconsistent with these risk factors, 50% of WD patients with LC in our cohort were female. Bandmann et al. proposed that neurologic deterioration in some patients may be caused by excessive mobilization of copper during chelator therapy, leading to an increase in the toxic “free” copper pool [41]. This hypothesis is supported by a pivotal study by Brewer et al., demonstrating the correlation between elevated non‐ceruloplasmin‐bound copper and worsening neurologic outcomes [51]. Therefore, the increased LC prevalence and mental health impact in WD patients may be due to the chronic liver disease and elevated copper levels [3, 51, 52, 53, 54], though this is not limited to those with known neuropsychiatric manifestations. Within our cohort of WD patients, onset of symptoms was significantly earlier in primarily hepatic patients than in those with primarily neurologic/psychiatric or mixed manifestation. Interestingly, 85% of the WD patients with LC suffered from primarily hepatic manifestation, whereas only 62% of the WD patients without LC suffered from primarily hepatic manifestation. Therefore, a potential influence of earlier onset of symptoms—and a potential longer toxic copper influence‐ in WD might play an important role for the occurrence of LC in these patients. Other studies suggest that comorbidities increase susceptibility to neurocognitive decline [47]. Our study suggests a higher LC prevalence in WD patients 11 months post‐infection compared to studies on mild, non‐hospitalized SARS‐CoV‐2 cases [15, 16, 55]. WD patients, already presenting with a wide range of symptoms, are more likely to develop LC, though LC may only affect a subset of both WD patients and the general population [13].

Over half of WD patients recover without long‐term impacts on quality of life or mental health, despite having a chronic disease. The LC subgroup had a median age of 33 years, compared to 39 years for the full cohort, with a similar median time since WD diagnosis (22 years vs. 23 years). Neurological symptoms were present in 25% of the LC subgroup (vs. 29.4% in the full cohort), and 15% had compensated liver cirrhosis (vs. 13.7%). Treatment with trientine was more common in the LC subgroup (80%) compared to recovered patients (63.3%), while d‐penicillamine use was lower in the LC group (15% vs. 38.7%). Despite the high proportion of trientine‐treated WD patients, these differences were not statistically significant. Similar to the general population, vaccination did not prevent LC, as all LC patients were fully vaccinated before their first infection. Vaccination rates in the cohort were high, with 86.3% completing both doses at least two weeks before infection and 92.2% receiving both doses overall, consistent with previous studies on vaccination adherence in WD patients [49]. Comparing genotype vs. phenotype correlation, studies suggest that a homozygous mutation for H1069Q, compared to H1069Q compound heterozygous patients, leads to a later onset of disease but more frequent neurological manifestations [5, 42, 56]. In our cohort, 41.7% with homozygous mutation for H1069Q suffered from a neurological or mixed phenotyp. However, this observation could not be confirmed in larger studies [57]. In our data, a correlation between genotype and initial phenotype/occurrence of LC could not be clearly defined. Nevertheless, 85% of LC WD patients suffered from hepatic phenotype. Our study has several limitations. WD is a rare genetic disorder, and the small sample size may limit statistical power. Data were collected retrospectively via a self‐conducted survey, and the effects of the SARS‐CoV‐2 infection may be influenced by broader pandemic‐related factors, such as increased morbidity, mental health challenges, economic disruptions, and social isolation [46, 47]. Pandemic effects also encompass economic disruptions such as job losses, business closures and a global recession, along with social consequences like isolation, educational disruptions and exacerbated inequalities [58, 59]. Additionally, the pandemic has led to significant behavioral changes, such as increased remote work, heightened hygiene practices and has strained healthcare systems while accelerating digital transformation [60]. Thus, we cannot exclude a contribution of any of these effects and subsequent psychological impact on the WD cohort.

Nevertheless, this is the first study to analyze LC in WD and its mental health effects. Our findings show that many WD patients experience long‐term symptoms and mental health deterioration after SARS‐CoV‐2 infection. WD patients should be closely monitored post‐infection, with attention to the potential negative impact on treatment adherence. Comprehensive therapies like physiotherapy, ergotherapy, and psychological or neurological evaluations may be needed and should be implemented in clinical routine. This study will help clinicians to devise better and react more appropriately among therapeutic decisions. Further research is required to understand the long‐term cognitive effects of COVID‐19 in WD and its potential impact on liver disease, particularly in patients with cirrhosis. Our data underscore the need for further investigations into the long‐term cognitive effects of COVID‐19 infection in WD and also the effect of SARS‐CoV‐2 on the development of the underlying liver disease in WD, with special interest in patients with cirrhosis.

## 5 Conclusion

COVID‐19 infection primarily affects physical health, sleep disturbances, and fatigue in WD patients. LC prevalence is higher than in the general population, with significant mental health impacts on quality of life, sleep, fatigue, depression, anxiety, stress, and cognitive complaints. An earlier onset of symptoms in WD might play an important role in the occurrence of LC in these patients. In summary, a large proportion of Wilson disease patients experience long‐term persistent symptoms and deteriorated mental health after SARS‐CoV‐2 infection. Therefore, Wilson disease patients should be well monitored post‐infection, especially considering the potential negative impacts of reduced mental health on adherence, which is essential for reaching desired therapeutic goals. Further studies are needed to examine the impact of SARS‐CoV‐2 on the natural course of WD.

## Author Contributions

Study concept and design: Isabelle Mohr. Acquisition, analysis, and interpretation of the data: Maximilian Brand, Christophe Weber, Patrick Michl, Andrea Langel, Jessica Langel, and Viola Yuriko Leidner. Drafting the article: Isabelle Mohr and Uta Merle. Critical revision for important intellectual content: Isabelle Mohr, Maximilian Brand, Christophe Weber, Patrick Michl, Viola Yuriko Leidner, Alexander Olkus, Sebastian Köhrer, and Uta Merle. Statistical analysis: Isabelle Mohr and Maximilian Brand. Study supervision: Uta Merle. All authors approved the final version of the article and agreed to be accountable for all aspects of the work.

## Ethics Statement

All procedures followed were in accordance with the ethical standards of the responsible committee on human experimentation (institutional and national) and with the Helsinki Declaration of 1975, as revised in 2000 (5). Ethics approval was obtained (University of Heidelberg, protocols S‐565/2011 and S‐546/2020), and all participants provided written informed consent. Proof of informed consent is available upon request from the corresponding author [**I**sabelle Mohr].

## Consent

Written informed consent for the procedure and management was obtained from all individual participants included in the study.

## Conflicts of Interest

**I**sabelle Mohr advises for Univar, received travel grants from Univar and received speaker's fees from Orphalan. Maximilian Brand, Christophe Weber, Andrea Langel, Jessica Langel, Viola Yuriko Leidner, Alexander Olkus, and Sebastian Köhrer declare no conflicts of interest. Patrick Michl received honoraria from Falk, Orphalan, and AstraZeneca. Uta Merle received honoraria for teaching from Falk and Univar and received travel grants from Gilead and Falk.

## Supporting information


**Data S1.** Supporting Information.

## Data Availability

Data are available from the authors with the permission of the corresponding author. The data that support the findings of this study are available from the corresponding author [**I**sabelle Mohr] upon reasonable request.
