# Supplementary material for: Mental and Physical Health in Wilson Disease Patients With SARS‐CoV‐2 Infection and Relevance of Long‐COVID
Source: JIMD Rep. 2025 May 6;66(3):e70021. doi: 10.1002/jmd2.70021 (PMC12055521; doi:10.1002/jmd2.70021)
Supplement: Supplementary file 1 — Data S1. Supporting Information. [file JMD2-66-e70021-s001.docx]

**Mental and physical health in Wilson disease patients with SARS-CoV-2 infection and relevance of Long-COVID**

**Supplemental material**

**Isabelle Mohr^1^, Maximilian Brand^1^, Christophe Weber^2^, Andrea Langel^1^, Jessica Langel^1^, Patrick Michl^1^, Viola Yuriko Leidner^1^, Alexander Olkus^1^, Sebastian Köhrer^1^, Uta Merle^1^**

^1^Internal Medicine IV, Department of Gastroenterology, University Hospital Heidelberg, Heidelberg, Germany

^2^Internal Medicine III Department of Internal Medicine and Cardiology, University Hospital Heidelberg, Heidelberg, Germany

**Corresponding author:**

Dr. med. Isabelle Mohr

Internal Medicine IV

Department of Gastroenterology, University Hospital Heidelberg

Im Neuenheimer Feld 410
69120 Heidelberg
Telefon: +49 6221 56 32818
Telefax: +49 6221 56 5694
eMail: [isabelle.mohr@med.uni-heidelberg.de](mailto:isabelle.mohr@med.uni-heidelberg.de)

**Key words:** Wilson disease, COVID-19, SARS-CoV-2 infection, Long COVID, mental health, quality of life

**Supplemental Table 1: Summary of symptoms for patients with long COVID symptoms (n=20) versus recovered group (n=31)**

| **New Symptoms after COVID-19 infection in WD Patients** | **Long COVID subgroup n (%)**  **n=21** | **Recovered subgroup n (%)**  **n=31** | **Chi-Quadrat Test (p=value)** |
| --- | --- | --- | --- |
| **1 Cough** | 5 (25) | 7 (22) | 0.842 |
| **2 Fever** | 0 (0) | 0 (0) | NA |
| **3 Shortness of breath**   1. **at rest** 2. **mild activity** 3. **extended acitivity** | 8 (40) | 3 (10) | **<0.001** |
|  | 1 (5) | 0 (0) | 0.750 |
|  | 8 (40) | 1 (3) | **<0.001** |
|  | 8 (40) | 2 (6) | **0.003** |
| **4 Palpitation** | 3 (15) | 3 (10) | 0.565 |
| **5 Chest pain**   1. **at rest** 2. **extended acitivity** | 4 (20) | 1 (3) | **0.049** |
|  | 2 (10) | 0 (0) | 0.315 |
|  | 4 (20) | 1 (3) | **0.049** |
| **6 Fatigue** | 11 (55) | 8 (26) | **0.035** |
| **7 Myalgia** | 6 (30) | 4 (13) | 0.133 |
| **8 Athralgia** | 3 (15) | 5 (16) | 0.914 |
| **9 Muscular weakness** | 7 (35) | 7 (22) | 0.332 |
| **10 Cephalgia** | 11 (55) | 6 (19) | **0.008** |
| **11 Vertigo** | 9 (45) | 5 (16) | **0.024** |
| **12 Feeling of brain fog** | 3 (15) | 5 (16) | 0.914 |
| **13 Anxieties** | 3 (15) | 6 (19) | 0.690 |
| **14 Depression** | 3 (15) | 7 (22) | 0.506 |
| **15 Sleep disturbances** | 7 (35) | 7 (22) | 0.332 |
| **a) falling asleep** | 5 (25) | 8 (26) | 0.949 |
| **b) sleeping through** | 7 (35) | 4 (13) | 0.061 |
| **16 Stool disturbances** | 5 (20) | 2 (6) | 0.060 |
| 1. **diarrhea** | 3 (15) | 1 (3) | 0.127 |
| 1. **more obstipation** | 2 (10) | 1 (3) | 0.315 |
| **17 Dysgeusia** | 4 (20) | 0 (0) | **0.009** |
| **18 Dysosmia** | 1 (5) | 1 (3) | 0.750 |
| **19 Alopecia** | 3 (15) | 4 (13) | 0.832 |
| **20 Sore throat** | 7 (35) | 0 (0) | **< 0.001** |
| **21 Rhinitis** | 9 (45) | 0 (0) | **< 0.001** |
| **22 Exercise capacity** | 10 (50) | 4 (13) | **0.004** |
| **23 Blood pressure variations** | 4 (20) | 1 (3) | 0.050 |
| **24 Concentration disorders** | 12 (60) | 4 (13) | **< 0.001** |
| **25 Amnestic dysphasia** | 3 (15) | 0 (0) | **0.026** |
| **26 Memory disturbances** | 5 (20) | 2 (6) | 0.060 |

Legend: NA= not available

**Supplemental Table 2: Summary of questionnaire of symptoms before vs. after SARS-CoV-2 infection**

| Symptom | **Before COVID-19** | | | | | **At questionnaire timepoint (after acute COVID-19)** | | | | |
| --- | --- | --- | --- | --- | --- | --- | --- | --- | --- | --- |
| n=51 (100%) with COVID-19 infection | n | n | If **yes (n)**: | | |  |  | If **yes (n):** | | |
|  | **no** | **yes** | mild | moderate | severe | **no** | **yes** | mild | moderate | severe |
| **1 Cough** | 49 | 2 | 2 | 0 | 0 | 39 | 12 | 7 | 5 | 0 |
| **2 Fever** | 51 | 0 | 0 | 0 | 0 | 51 | 0 | 0 | 0 | 0 |
| **3 Shortness of breath**   1. **at rest** 2. **mild activity** 3. **extended acitivity** | 45 | 6 | 1 | 2 | 3 | 40 | 11 | 7 | 4 | 0 |
|  |  | 1 | 1 | 0 | 0 |  | 2 | 1 | 1 | 0 |
|  |  | 2 | 0 | 2 | 0 |  | 9 | 6 | 3 | 0 |
|  |  | 3 | 0 | 0 | 3 |  | 10 | 2 | 7 | 1 |
| **4 Palpitation** | 45 | 6 | 5 | 0 | 1 | 45 | 6 | 4 | 2 | 0 |
| **5 Chest pain**   1. **at rest** 2. **extended acitivity** | 49 | 2 | 1 | 1 | 0 | 46 | 5 | 4 | 0 | 1 |
|  |  | 1 | 1 | 0 | 0 |  | 2 | 1 | 1 | 0 |
|  |  | 1 | 0 | 1 | 0 |  | 5 | 3 | 1 | 1 |
| **6 Fatigue** | 34 | 17 | 13 | 4 | 0 | 32 | 19 | 8 | 10 | 1 |
| **7 Myalgia** | 44 | 7 | 5 | 1 | 1 | 41 | 10 | 4 | 4 | 2 |
| **8 Athralgia** | 43 | 8 | 6 | 1 | 1 | 43 | 8 | 5 | 1 | 2 |
| **9 Muscular weakness** | 41 | 10 | 4 | 5 | 1 | 37 | 14 | 6 | 6 | 2 |
| **10 Cephalgia** | 45 | 6 | 4 | 2 | 0 | 34 | 17 | 9 | 5 | 3 |
| **11 Vertigo** | 45 | 6 | 4 | 2 | 0 | 37 | 14 | 12 | 2 | 0 |
| **12 Feeling of brain fog** | 44 | 7 | 3 | 3 | 1 | 43 | 8 | 4 | 2 | 2 |
| **13 Anxieties** | 43 | 8 | 5 | 3 | 0 | 42 | 9 | 5 | 3 | 1 |
| **14 Depression** | 42 | 9 | 7 | 1 | 1 | 41 | 10 | 7 | 3 | 0 |
| **15 Sleep disturbances** | 35 | 16 | 13 | 3 | 0 | 37 | 14 | 6 | 7 | 1 |
| **a) falling asleeep**  **b) sleeping through** |  | 14 | 10 | 4 | 0 |  | 13 | 4 | 9 | 0 |
|  |  | 8 | 7 | 1 | 0 |  | 11 | 5 | 4 | 2 |
| **16 Stool disturbances** | 47 | 4 | 2 | 2 | 0 | 44 | 7 | 4 | 2 | 1 |
| 1. **diarrhea** |  | 1 | 1 | 0 | 0 |  | 4 | 2 | 2 | 0 |
| 1. **more obstipation** |  | 3 | 1 | 2 | 0 |  | 3 | 2 | 1 | 0 |
| **17 Dysgeusia** | 51 | 0 | 0 | 0 | 0 | 47 | 4 | 3 | 1 | 0 |
| **18 Dysosmia** | 50 | 1 | 1 | 0 | 0 | 49 | 2 | 2 | 0 | 0 |
| **19 Alopecia** | 46 | 5 | 3 | 1 | 1 | 44 | 7 | 2 | 4 | 1 |
| **20 Sore throat** | 49 | 2 | 2 | 0 | 0 | 44 | 7 | 3 | 3 | 1 |
| **21 Rhinitis** | 47 | 4 | 3 | 1 | 0 | 42 | 9 | 5 | 4 | 0 |
| **22 Exercise capacity** | 43 | 8 | 3 | 4 | 1 | 37 | 14 | 7 | 6 | 1 |
| **23 Blood pressure variations** | 48 | 3 | 2 | 1 | 0 | 46 | 5 | 4 | 1 | 0 |
| **24 Concentration disorders** | 39 | 12 | 9 | 3 | 0 | 35 | 16 | 7 | 7 | 2 |
| **25 Amnestic dysphasia** | 38 | 13 | 10 | 3 | 0 | 39 | 12 | 6 | 5 | 1 |
| **26 Memory disturbances** | 43 | 8 | 7 | 1 | 0 | 44 | 7 | 2 | 5 | 0 |

**Supplemental Table 3: Phenotype and Genotype correlation within the WD cohort**

| Nr. | Long  Covid | Mutation 1 | Mutation 2 | Manifestation Phenotype at initial diagnosis | Age at Diagnosis | Gender |
| --- | --- | --- | --- | --- | --- | --- |
|  | yes | R1041W | H1069Q | hepatic | 16 | male |
|  | yes | D765N | M1169V | hepatic | 8 | female |
|  | yes | H1069Q | 3400delC | hepatic | 8 | Male |
|  | yes | M769H-fs | H1069Q | hepatic | 9 | Female |
|  | yes | H1069Q | H1069Q | Hepatic | 38 | Female |
|  | yes | H1069Q | H1069Q | Mixed | 17 | Female |
|  | yes | H1069Q | H1069Q | Hepatic | 13 | Male |
|  | yes | K832R | K832R | Hepatic | 15 | Female |
|  | yes | V1217121 | V1217121 | Hepatic | 5 | Male |
|  | yes | G1405S | H1069Q | Hepatic | 21 | Female |
|  | yes | H1069Q | 2299InsC | Hepatic | 50 | Male |
|  | yes | G1030S | H1069Q | Hepatic | 18 | Female |
|  | yes | Q1351X | W779X | Hepatic | 25 | Male |
|  | yes | H1069Q | H1069Q | Neurological | 14 | Male |
|  | yes | T858A | T858A | Hepatic | 27 | Male |
|  | yes | K844K-fs | K844K-fs | Mixed | 20 | Female |
|  | yes | A1140V | H1069Q | Hepatic | 10 | Male |
|  | yes | W779X | W779X | Hepatic | 15 | Female |
|  | yes | IVS12-1: | IVS12-1: | Hepatic | 16 | Female |
|  | yes | H1069Q | W779X | Hepatic | 25 | Male |
|  | no | H1069Q | H1069Q | Mixed | 30 | Female |
|  | no | R1041W | R1041W | Mixed | 3 | Male |
|  | no | H1069Q | H1069Q | Hepatic | 16 | Female |
|  | no | M665I | W779X | Hepatic | 36 | Female |
|  | no | H1069Q | R969Q | Hepatic | 16 | Male |
|  | no | H1069Q | H1069Q | Hepatic | 17 | Female |
|  | no | H1069Q | H1069Q | Mixed | 29 | Male |
|  | no | W779X | H1069Q | Hepatic | 32 | Male |
|  | no | H1069Q | H1069Q | Neurological | 6 | Female |
|  | no | H1069Q | H1069Q | Hepatic | 6 | Male |
|  | no | L770P | H1069Q | Hepatic | 26 | Female |
|  | no | H1069Q | H1069Q | Hepatic | 11 | Male |
|  | no | R969Q | H1069Q | Hepatic | 17 | Male |
|  | no | H1069Q | H1069Q | Hepatic | 25 | Female |
|  | no | H1069Q | H1069Q | Neurological | 8 | Female |
|  | no | Q1351X | H1069Q | Hepatic | 33 | Female |
|  | no | H1069Q | R969Q | Neurological | 16 | Male |
|  | no | S1067N | L667X | Hepatic | 31 | Female |
|  | no | H1069Q | H1069Q | Neurological | 5 | Female |
|  | no | H1069Q | H1069Q | Mixed | 15 | Female |
|  | no | H1069Q | H1069Q | Hepatic | 16 | Female |
|  | no | H1069Q | H1069Q | Hepatic | 17 | Female |
|  | no | H1069Q | D765H | Hepatic | 19 | Female |
|  | no | H1069Q | W779X | Mixed | 11 | Male |
|  | no | H1069Q | H1069Q | Hepatic | 14 | Male |
|  | no | H1069Q | H1069Q | Hepatic | 18 | Female |
|  | no | R969Q | Y1078Y-f | Mixed | 16 | Female |
|  | no | H1069Q | R969Q | Neurological | 37 | Male |
|  | no | H1069Q | P1273L | Hepatic | 13 | Female |
|  | no | 2299InsC | H1069Q | Neurological | 14 | Male |
|  | no | H1069Q | H1069Q | Hepatic | 10 | female |
